# Catchment Influences on Carbon Stable Isotope Variation in Trout; Might It Be Methane?

**DOI:** 10.1002/ece3.73554

**Published:** 2026-04-29

**Authors:** Michael Hinchliffe, Aimeric Blaud, Peter Gilbert, Rona McGill, Kenny Galt, Robert A. Briers

**Affiliations:** ^1^ Edinburgh Napier University, School of Applied Sciences, Edinburgh Napier University Edinburgh UK; ^2^ Centre for Conservation and Restoration Science, Edinburgh Napier University Edinburgh UK; ^3^ University of Highlands and Islands Environmental Research Institute Thurso UK; ^4^ Royal Society for the Protection of Birds (RSPB), Etive House Inverness UK; ^5^ Stable Isotope Ecology Lab, Natural Environment Isotope Facility, Scottish Universities Environmental Research Centre East Kilbride UK; ^6^ Tweed Foundation Drygrange Steading Melrose UK

**Keywords:** food web, land use, methane derived carbon, salmonid, soil drainage, stream

## Abstract

Stable isotope analysis is widely used in ecology to trace spatial origins and trophic interactions for many species. Salmonid fish, including brown trout ( *Salmo trutta* ), are often a focal species for stable isotope analysis due to the high ecological importance and anadromous life cycles. Variations in δ^13^C values of trout have been linked to catchment characteristics, such as land use. These variations reflect carbon processes at the base of the food web, with chemosynthetic processes such as methane oxidation producing lower values than those of the photosynthetic carbon processing. We examined δ^13^C values in brown trout fry across 51 sites in two tributaries of the River Tweed, Scotland to investigate catchment drivers of trout δ^13^C variation. Soil drainage and pasture cover were identified as the strongest predictors of fry δ^13^C values, with lower drainage and higher pasture correlating with lower δ^13^C. A subset (26) of the sites was sampled for mayflies (*Baetis* spp.), finding a strong correlation between the δ^13^C values of mayflies and trout fry, suggesting trout δ^13^C is indicative of broader carbon processes at the site. Increases in cover of pasture and low drainage soils are known to result in elevated CH_4_ concentrations in streams, while low δ^13^C values are an indicator of methane‐derived carbon in the food web. The observed link between δ^13^C variation and catchment features associated with methane production, such as poorly drained soils and pasture, points to a potential role for methane‐derived carbon in structuring upland stream food webs.

## Graphical abstract

Trout stable carbon isotope values vary significantly and appear to relate to catchment characteristics. Low carbon stable isotope values are linked to catchments with low drainage soil and high cover of pasture land use. This is likely to be linked to high levels of methane‐derived carbon entering the stream food web.

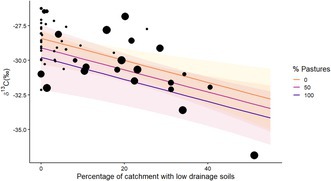

## 1 Introduction

The study of stable isotopes has become an important tool in the ecologist's arsenal. The knowledge of how different elements change with metabolic processes or how they vary with geographic region has led to their use in tracking migratory species (Hobson 1999; McCarthy and Waldron 2000; Trueman et al. 2012), investigating wildlife crimes (Prigge et al. 2025), tracing fishing stocks (Llorente‐Rodríguez et al. 2022) and constructing accurate food webs (Ben‐David and Schell 2001; Post 2002). Carbon fixation forms the basis of all food webs, either through processes such as photosynthesis, decomposition, or chemosynthesis, or a mixture of the three (Pusch et al. 1998; Brett et al. 2017). As each metabolic process selects for ^12^C and ^13^C with varying degrees of discrimination, the ratio between these carbon isotopes, known as δ^13^C, can be used to track changes in which carbon fixation processes support food webs.

In riverine food webs, δ^13^C values of the primary consumers are often dependent on their feeding niche and the relative availability of basal carbon sources. Terrestrial inputs such as particulate organic matter and detritus usually have values around −25‰ to −30‰ (Adams et al. 2015). Carbon fixed via photosynthesis from macrophytes and algae typically ranges from −35‰ to −10‰ (Kohn 2010; Messerschmid et al. 2021), while methane oxidation, the main chemosynthetic carbon pathway, ranges from −50‰ to −110‰ (Hornibrook et al. 2000; Michener and Lajtha 2008; Rulík et al. 2013; Zazzeri et al. 2016). As such, primary consumers' δ^13^C values reflect a mix of these values depending on the carbon sources utilised, with local scale variation in a species’ δ^13^C value indicating wider trophic niches and large spatial scales indicating changes in available basal resources (Bearhop et al. 2004; Potapov et al. 2019).

The distinctly low δ^13^C values from methane derived carbon (MDC) make it easier to identify individuals with substantial carbon contributions from methane (Grey 2016). In lotic systems, MDC has been discovered in primary consumers such as Chironomidae (Jones et al. 2008; Jones and Grey 2011; Agasild et al. 2014) and predatory fish such as ruffe (*Gymnocephalus cernua*, δ^13^C ranging from −43.6‰ to −26.1‰) (Ravinet et al. 2010), Japanese eel (*Anguilla japonica*, −37.95‰ to −20.88‰) (Tsuchiya et al. 2024) and apex predators such as pike (*Esox lucius*, ~ −32‰) (Agasild et al. 2014). In riverine systems, the evidence for MDC has mostly been described in macroinvertebrates, in particular caddisflies (Sampson et al. 2019), where δ^13^C values were seen to vary with catchment geology, a factor known to influence riverine CH_4_ concentrations (Jones and Mulholland 1998). Both CH_4_ concentrations and the abundance and composition of methanotrophs, the microbial vector through which methane becomes available to the broader food web (Bodelier et al. 2019), have been shown to vary with catchment conditions, in particular temperatures and land use (Stanley et al. 2016; Nagler et al. 2020). Anthropogenic land use, such as urbanisation and agriculture, is thought to increase CH_4_ concentrations, alongside other geological drivers such as soil type (He et al. 2024) and drainage (Shukla et al. 2013), floodplain connectivity (Machado dos Santos Pinto et al. 2020) and the presence of peatland (Hope et al. 2001; Stanley et al. 2016). As such, these factors may influence MDC incorporation and could contribute to lower δ^13^C values in riverine consumers. At the same time, catchment characteristics will alter other carbon inputs, such as terrestrial organic matter (Masese et al. 2017) and light availability for in‐stream photosynthesis (Julian et al. 2008; Bowes et al. 2012), leading to shifts in the carbon isotope characteristics within the stream food web (Campeau et al. 2018; Nkoue Ndondo et al. 2021).

In this study, we investigated spatial variation in brown trout fry isotope values across headwater streams of the River Tweed, Scotland. In addition to trout fry, *Baetis* sp., a common primary consumer, was also sampled at a subset of sites to test if trout fry isotopes reflect changes in basal resources. We aimed to test three main hypotheses: (1) Trout fry δ^13^C values will vary spatially among sites, and lower values will be associated with catchment characteristics known to correlate with higher riverine CH_4_ concentrations. (2) Trout fry δ^13^C values will reflect site‐level functioning by correlating with the δ^13^C of a common primary consumer, *Baetis*. (3) Links between δ^13^C variation in trout fry and catchment characteristics will vary with spatial scale, as a result of relevant processes acting at different scales.

## 2 Materials and Methods

### 2.1 Study Species *Salmo trutta*

Brown trout were chosen as a study species due to their near ubiquitous distribution within the study system as well as the relatively low and variable carbon stable isotope values found in previous studies such as Briers et al. (2013). The majority of the diet of brown trout fry is thought to be comprised of Chironomidae spp. and Baetidae spp. (Sánchez‐Hernández et al. 2011), both of which have shown isotopic values thought to reflect MDC incorporation (Jones et al. 2008; Yasuno et al. 2013; Pearson 2015), suggesting a plausible link between the carbon in *S. trutta* fry and MDC.

### 2.2 Sample Collection and Stable Isotope Analysis

**FIGURE 1:**
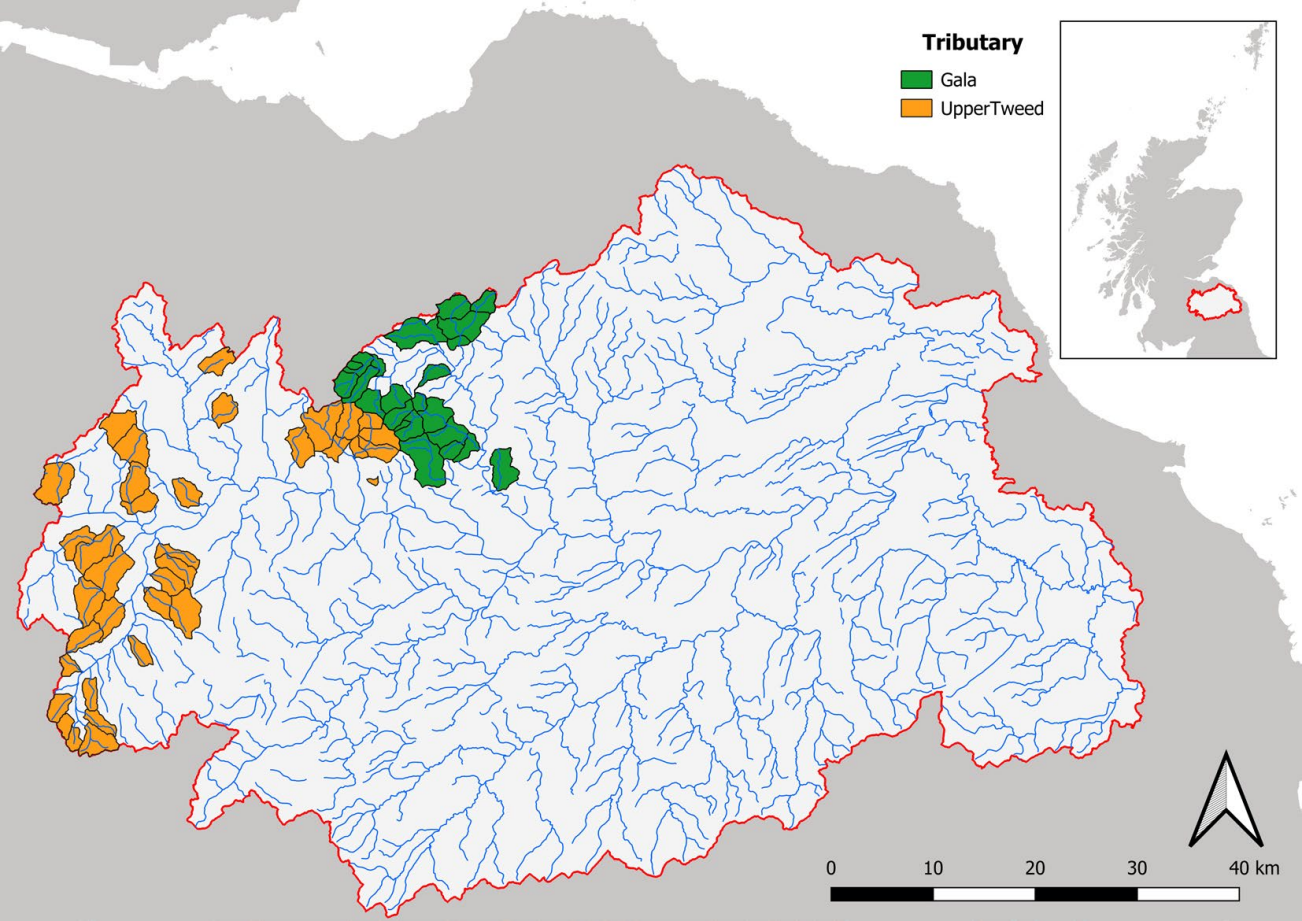
Map of the Tweed catchment. The sites within the Gala Water (orange) and the Upper Tweed (green) tributaries are pictured, with each outlined polygon depicting a subcatchment derived from the point where the trout fry were captured. Crown copyright and database rights 2025 Ordnance Survey (100025252).

Brown trout fry were obtained from samples collected between mid‐August and mid‐September 2012 as part of fishery management surveys by staff from the Tweed Foundation (the fisheries management organisation for the River Tweed); as a result no additional ethical approval was required (confirmed by Edinburgh Napier Research Ethics Committee). Samples were obtained from 51 sites across two tributaries of the Tweed catchment; the Upper Tweed and the Gala Water (Figure 1), using backpack electrofishing gear and euthanised with an overdose of anaesthetic. Sampling at each site was constrained to a short stretch of the stream (~20 m), with sampling commencing until 10 fry were collected. Timing of sampling gave any anadromous fry at least 6 months of freshwater feeding to equilibrate to the isotopic signature of the stream (Briers et al. 2017). Due to the size of the fry sampled (range of 21–74 mm in length) and previous literature, they were presumed to have been feeding on invertebrates in close proximity to their sample point (Foldvik et al. 2010; Kvingedal and Einum 2011). However, drift from upstream invertebrates and movement of fry cannot be ruled out. Following sampling, the fry were kept in field cool boxes at around 4°C, and then kept frozen at −20°C prior to processing. Individual fish data can be seen in S1.

For stable isotope analysis, white muscle tissue was dissected from behind the dorsal fin of the fish from each site. The dissected area was kept consistent as fish tissues are known to be highly variable in carbon and nitrogen stable isotope values (Hayden et al. 2015). Muscle samples were dried individually for 24 h at 60°C in a drying oven before weighing out a mass of 0.7 ± 0.1 mg into tin capsules for analysis. Lipid correction was not carried out at this stage due to the presumptions that fry of this size would contain relatively low lipid concentrations; this was further supported by C:N ratio, with all fry samples found to have a ratio of ~3.5, indicating that the lipid concentration would not need correcting for (Post et al. 2007).

The samples were then analysed for their carbon and nitrogen stable isotope compositions at the National Environmental Isotope Facility ecology laboratory in East Kilbride. The analysis involved continuous flow isotope ratio mass spectrometry (CF‐IRMS), using an Elementar Pyrocube Elemental Analyser interfaced with a ThermoFisher Scientific Delta XP Plus IRMS. Stable isotope ratios were expressed as ‰ relative to a standard (Pee Dee Belemnite for δ ^13^C, atmospheric air for δ^15^N). The standard deviation of multiple analyses of an internal gelatine standard was less than 0.1‰ for both δ^13^C and δ^15^N. For all statistical analyses outside of site variation, the mean stable isotope value of the 10 fish samples was calculated for each site.

To assess if the isotopic values seen within the trout reflect variation in lower trophic levels, a known abundant prey taxon, *Baetis*, was sampled alongside the trout for their carbon isotopic values. *Baetis* spp. were collected in a subset of 26 of the sample sites (7 in the Gala and 19 in the Upper Tweed) using a standard kick‐net (1 mm mesh, 250 mm width). *Baetis* were prepared in a similar way as the trout muscle tissue samples. Each *Baetis* was individually dried for 24 h at 60°C in a drying oven before being homogenised and a mass of 0.7 ± 0.2 mg from each *Baetis* was placed into tin capsules. Where an individual was below the 0.5 mg threshold for reliable analysis, multiple *Baetis* were combined to reach the required weight. No lipid correction was applied for consistency with the process for trout fry.

### 2.3 Deriving the Sub‐Catchments

To define the catchment upstream of each sampling point the Ordnance Survey Terrain 5 digital elevation model (scale 1:10,000) was used in conjunction with hydrological and watershed tools found within Whitebox Geospatial Analysis Tools (version 1.0.2) (Lindsay 2016). The GPS locations of each sample site were snapped to the nearest stream segment (within 50 m) and the boundaries of the upstream catchment of each sample point were derived. Although none of the catchments of the sampling sites included large still water sections it is not possible to totally rules out the movement of trout fry from these areas.

### 2.4 Extracting Catchment Characteristics

The digital elevation model described above was used to derive the mean elevation and mean slope gradient for each sub catchment, using tools within QGIS version 3.16.0 (QGIS Development Team 2023). The land uses for each catchment were extracted using the CORINE land cover inventory for 2012 (Cole et al. 2015). Land use types not well‐represented (< 1% of total area from all catchments) were removed from further analysis as they could not be effectively evaluated in the analysis; these included: broad‐leaf forest, mixed forest and discontinuous urban fabric.

Soil drainage classification was extracted from The James Hutton Institute's 1:25,000 Soil Map Phase Seven database (Soil survey of Scotland staff 2022). For each catchment, the percentage area for each land use, drainage type, and soil type was derived. Soil drainage types were grouped into low, mixed, and high drainage soils for easier comparison. The grouping was done based on their attribution from the Phase Seven database with Low drainage soils encompassing: very poorly drained and poorly drained, Mixed drainage: undifferentiated drainage class and freely drained below the iron pan while High drainage consisted only of freely drained soils.

### 2.5 Data Analysis

All statistical analysis was completed using R version 4.2.2 (R Core Team 2022).

#### 2.5.1 Trout and *Baetis* Stable Isotope Values

Initial analysis was undertaken to determine whether carbon stable isotope ratio values were related to length of fry. Although there was a significant negative relationship, this only accounted for 0.2% of the variation in δ^13^C, so length was not considered a major influence on values and was not included in subsequent analyses.

Site variations in carbon and nitrogen stable isotope ratios for both the trout fry and *Baetis* were analysed using Kruskal‐Wallis for both trout and *Baetis* δ^13^C and trout δ^15^N values, while an analysis of variance (ANOVA) was used for *Baetis* δ^13^C values. The relationship between δ^13^C and δ^15^N for trout was assessed using Spearman rank correlation. Subsequent analysis focused on the carbon stable isotope values. A linear model was used to assess the relationship between the mean *Baetis* and trout stable δ^13^C values for the sites where both were sampled. Mean δ^13^C values of both trout and *Baetis* from each catchment were compared using *t*‐tests to determine if there was significant variation between the catchments, thus confirming that the two catchments were comparable and could be analysed together.

#### 2.5.2 Relationships Between Trout δ^13^C and Catchment Characteristics

The relationship between the trout δ^13^C values and catchment characteristics was assessed using linear models and a model selection‐based approach (Johnson and Omland 2004). A series of candidate models (details in Supporting Information S2) were constructed based on different additive combinations of the following variables: percentage of each of the four significant land use types within the catchment (coniferous forest, moors and heathland, pastures and transitional woodland scrub), percentage of the three drainage types, average altitude (m above sea level) of the catchment and average slope (degrees) of the catchment.

The combination of variables included in the model set reflected alternative hypotheses in relation to the influence of different land uses and thus carbon sources (see Introduction), with slope included based on its influence on catchment runoff characteristics and altitude owing to known temperature‐related influences on carbon isotope fractionation in soils (De Feudis et al. 2025).

Model selection was undertaken using the *MuMIn* package (version 1.43.17) (Bartoń 2026) in R, with selection based on the small sample Akaike Information Criterion (AICc). The differences between the AIC values of each model were calculated (ΔAIC) and a confidence set was derived from models whose ΔAIC < 2. Multicollinearity was checked for by calculating the variance inflation factor (VIF) of a global model, using the *car* package in R (Fox and Weisberg 2020). The highest VIF was 3.68; due to the relatively low VIF (Johnston et al. 2018), we considered multicollinearity between explanatory variables to not be an issue.

To assess the potential influence of spatial autocorrelation on the outcome of the model selection process, once the final averaged model was determined, an alternative was constructed which incorporated an exponential spatial error term based on sample site coordinates. Spatial and non‐spatial models were compared based on generalised least‐squares using the Restricted Maximum Likelihood model fitting and a Likelihood Ratio test in the *nlme* R package (Pinheiro et al. 2012) and no significant difference was found; hence, the non‐spatial model results are reported.

#### 2.5.3 Assessment of the Scale of Catchment Effects

**FIGURE 2:**
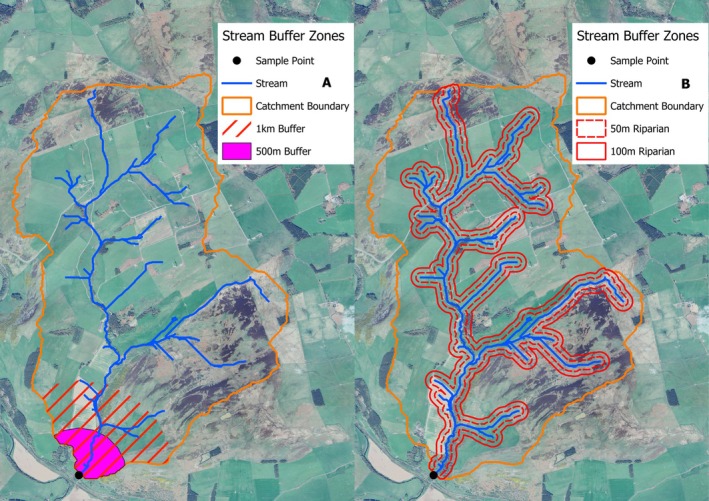
Example of a sub catchment from the Gala tributary (Satellite Map Data: Google, (c) 2024 This map includes data from: Airbus, Maxar Technologies) with buffers marked to show the different datasets compared. The white dot represents sampling point of the trout fry. The sub‐catchment boundary represents the entire area used when analysing for the whole catchment. (A) Shows the two buffer sizes set around the point of sampling. The red lines represent the area covered by the dataset from the 1 km buffer, while the purple fill represents the catchment area covered by the 500 m buffer. (B) Shows buffer sets based on the riparian zones of the stream. The red dotted line represents the area covered by the 50 m riparian buffer dataset and the black line represents the area covered by the 100 m riparian buffer dataset.

To evaluate how modelling from different catchment areas influenced the results, we considered four alternative catchment areas in addition to the overall sub‐catchment. Two buffers were created from the sample point and encompassed the area 500 m upstream and 1 km upstream, and two buffers encompassed the area 50 and 100 m either side of the entire watercourse within each catchment (Figure 2). For each buffer, the model selection process as described above was repeated and a confidence set of linear models was generated. The confidence sets of top models were then extracted and compared using their adjusted *R* ^2^ values as a measure of relative predictive power.

## 3 Results

### 3.1 Trout and *Baetis* Stable Isotope Variance and Relationship Between Values

**FIGURE 3:**
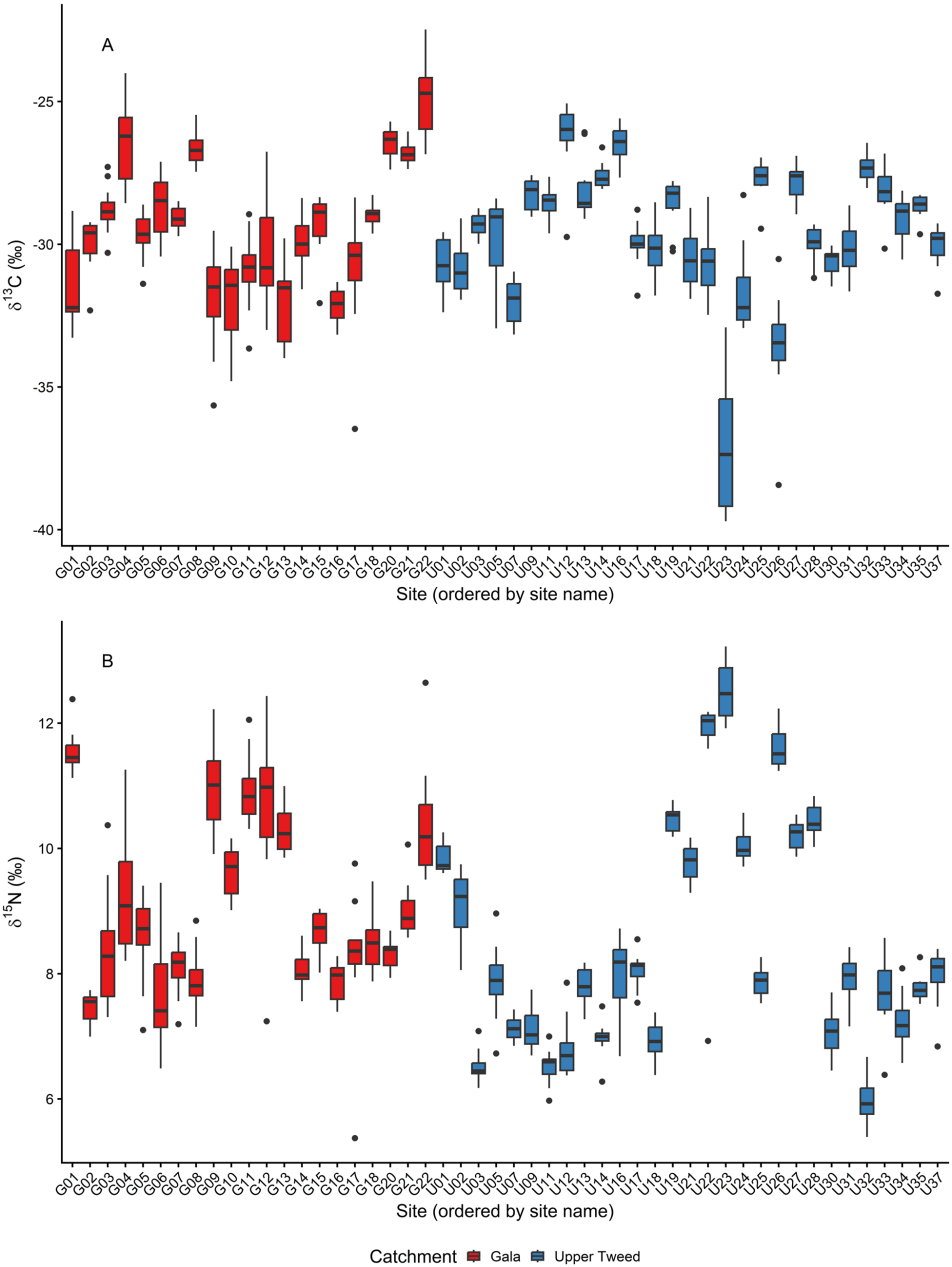
Brown trout fry ( *Salmo trutta* ) δ^13^C (A) and δ^15^N (B) values for each of the 51 sites sampled; 10 trout fry were sampled at each site. Blue boxes represent sites in the Upper Tweed tributary, red boxes represents Gala sites.

Individual trout fry δ^13^C values across all 51 sample sites ranged from −39.7‰ to −22.5‰ (S1) while δ^15^N ranged from 5.4‰ to 13.2‰ (Figure 3). Both δ^13^C (*χ* ^2^ = 473.27, df = 49, *p* < 0.001) and δ^15^N (*χ* ^2^ = 509.38, df = 49, *p* < 0.001) values showed significant variation between sites. There was a significant negative correlation between δ^13^C and δ^15^N values in trout fry (*r*_*s*_ = −0.27, *p* < 0.001).

*Baetis* were only sampled at a subset of 26 sites; δ^13^C values ranged from −43.3‰ to −26.3‰ (S1, S3) and δ^15^N from 1.3‰ to 10.1‰ (S1). Both δ^13^C (*F*_(25,20)_ = 14.81, *p* < 0.0001) and δ^15^N (*χ* ^*2*^ = 43.77, df = 25, *p* = 0.01) values showed significant variation between sites.

**FIGURE 4:**
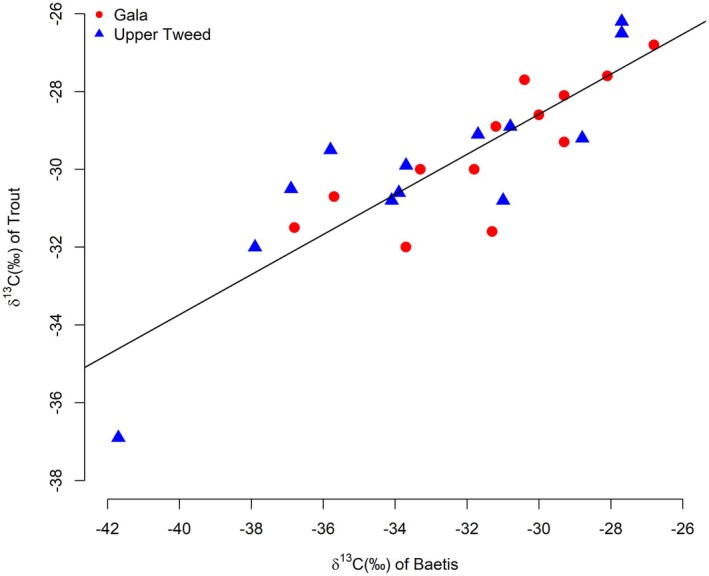
Linear regression of average trout fry δ^13^C plotted against average *Baetis* spp. δ^13^C. Tributaries are marked by different colours and shapes, Upper Tweed—Blue Triangles, Gala—Red circles.

The average δ^13^C value of trout showed a strong positive correlation with the average δ^13^C of *Baetis* spp. (*F*_(1,24)_ = 67.3, *p* < 0.0001, adjusted *R* ^*2*^ *= 0.73*) indicative of a strong trophic link between the two taxa (Figure 4). The δ^13^C value of the trout was on average 2.53‰ (range: 0‰ to 6.1‰) higher than the *Baetis*. There were no significant differences in the mean δ^13^C isotope values between catchments for either trout or *Baetis* (trout: *t* = 0.18, df = 12.8, *p* = 0.86; mean Gala = −29.63‰, mean Upper Tweed = −29.80‰; *Baetis*: *t* = 0.29, df = 8.6, *p* = 0.78; mean Gala = −32.69‰, mean Upper Tweed = −32.14‰).

### 3.2 Relationships Between Trout δ^13^C and Catchment Characteristics

A summary of the variation in the catchment characteristics being considered for model selection is given in Table 1.

**TABLE 1:** Summary of each catchment characteristic considered for model selection.

Category	Factor	Mean	Standard deviation	Range
Land use	Coniferous forest	10.38	21.49	0.00–90.96
Land use	Moors and Heathland	32.6	29.66	0.00–100.00
Land use	Pastures	16.15	22.75	0.00–64.34
Land use	Transitional Woodland Scrub	1.14	2.98	0.00–16.20
Drainage type	Low drainage	10.23	12.96	0.00–51.13
Drainage type	High drainage	48.8	24.81	11.90–90.06
Drainage type	Mixed drainage	17.05	14.5	0.00–51.69
Topography	Mean altitude	395.78	76.63	269.70–574.80
Topography	Mean slope	8.95	4.63	1.10–20.40

*Note:* Land use and drainage category values represent proportional catchment cover, altitude m above sea level and slope degrees. *N* = 51 for all factors.

**FIGURE 5:**
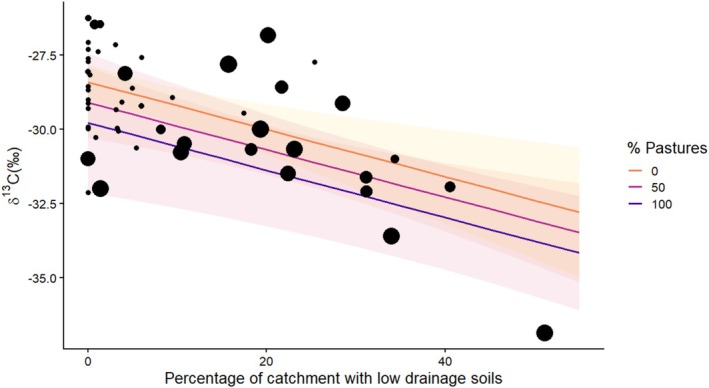
Plot of trout fry mean δ^13^C in relation to the percentage of the stream catchment that has low drainage soils. The percentage cover of pastures is represented by the size of the circles. Fitted lines indicate predicted relationships based on linear model output and shaded areas 95% confidence intervals.

For the catchment characteristics, only two models of the 31 tested had a ΔAIC < 2 and thus formed the confidence set (Table 2; Tables S1 and S2). These models contained combinations of percentage low drainage and pastures (Low drainage, Low drainage + Pastures), with δ^13^C values lower (indicating potential higher methane incorporation) with increasing cover of pastures and low drainage soils in the catchment (Figure 5).

**TABLE 2:** Summary of the variables included in the top performing models, with value of coefficient for each variable included after the variable name in parentheses.

Model number	Models	*k*	AICc	ΔAIC	Weight	*p*	Adjusted *R* ^2^
1	**Low drainage (−0.09)**	**1**	**198.6**	**0.00**	**0.366**	**3.34** × **10** ^**−6**^	**0.346**
2	**Low drainage (−0.08) + Pastures (−0.01)**	**2**	**199.6**	**0.99**	**0.223**	**1.195** × **10** ^**−5**^	**0.350**
3	Low drainage (−0.09) + Pastures (−0.02) + altitude (−0.005)	3	200.7	2.09	0.129	2.901 × 10^−5^	0.354
4	Low drainage (−0.09) + altitude (−0.008)	2	201.0	2.31	0.115	2.227 × 10^−5^	0.333
5	Low drainage (−0.08) + Pastures (−0.02) + slope (−0.06)	3	201.0	2.35	0.113	3.259 × 10^−5^	0.351
6	Low drainage (−0.09) + Pastures (−0.03) + Altitude (−0.004) + Slope (−0.04)	4	202.6	3.98	0.050	7.785 × 10^−5^	0.349
…25 models							
31	Transitional Wood Shrub (0.005) + Coniferous Forest (0.009)	2	223.1	24.45	< 0.001	0.747	−0.0291

*Note:* The top six models were included here, as the weighting dropped significantly. The bottom performing model (31) was also included for comparison. Our confidence set is formed of the two models highlighted in bold, selection was based on ΔAIC < 2. Full list of models displayed in Supporting Information S2.

### 3.3 Spatial Effects of Catchment Characteristics

Confidence sets across all five buffers considered included similar variables (Table 3), with pastures, low drainage types and altitude as the most frequent variables represented. Across all the buffers, the confidence sets from the whole catchment explained the highest variance (adjusted *R* ^2^ = 0.36–0.40) in the trout fry δ^13^C values (Table 3). The percentage of catchment area occupied by pastures was present in every confidence set and in every model. The highest proportion of the variation explained across all models included the interaction effect of pastures and low drainage soils (adjusted *R* ^2^ = 0.40; Table 3). Models in confidence sets from buffers with larger areas tended to have higher adjusted *R* ^2^ values (adjusted *R* ^2^ = 0.36–0.40), with the buffer from the smallest land area (500 m point) explaining the lowest amount of variance in δ^13^C values (adjusted *R* ^2^ = 0.14–0.18).

**TABLE 3:** Adjusted *R* ^2^ and significance levels for the confidence set of models from each buffer.

	LD	LD + PA	LD + PA + Al	LD + Al	PA	PA + MD	PA + Al	Al	Al + MH	PA + HD	PA + HD + Al	PA + MD + Al
Whole catchment	0.36***	0.38***	0.39***	—	—	—	—	—	—	—	—	—
1 km Point	—	0.24**	—	—	0.20***	0.22**	0.21**	—	—	—	—	—
500 m Point	—	0.17*	—	0.165*	0.15**	—	0.18**	0.14**	0.16*	—	—	—
100 m Riparian	—	0.24**	—	—	0.26**	—	—	—	—	0.25**	0.26**	0.26**
50 m Riparian	—	—	—	—	—	—	—	—	—	0.27***	0.28**	0.28**

*Note:* Significance denoted by *. *** < 0.001, ** < 0.01, * < 0.05. — indicates factors/models that were not included in the confidence set for each buffer size.

Abbreviations: Al, Altitude; HD, High drainage; LD, low drainage; MD, Mixed drainage; MH, Moors & Heathlands; PA, Pastures.

## 4 Discussion

### 4.1 Variation in δ^13^C Values and Potential Relation to MDC

There was significant variation in the δ^13^C of brown trout across the 51 sample sites, with ranges spanning > 15‰ (min −39.7‰, max −22.5‰) and also pronounced differences in the range of values evident at different sites. This large variation, both between and within sites, could be influenced by differences in carbon input from terrestrial or aquatic sources (Finlay et al. 2002; Abril and Borges 2019; Nkoue Ndondo et al. 2021) or variation in available prey species (Wang et al. 2019). Whilst the range of δ^13^C values in stream food webs can vary due to differences in the incorporation of terrestrial or photosynthetic carbon, the low δ^13^C values seen at many of the sites are in line with or below values previously noted as indicative of MDC contributions in freshwater fish (Ravinet et al. 2010; Sanseverino et al. 2012; Agasild et al. 2014; Tsuchiya et al. 2024; Urbano et al. 2025), suggesting that MDC, routed through lower parts of the food web, may form a significant part of the carbon stored in trout tissue.

Previous studies provide ample evidence that anthropogenic activities in the catchment can strongly influence both nutrient sources (carbon and nitrogen) and isotope ratios in aquatic food webs (e.g., Anderson and Cabana 2005; Al‐Nazzal et al. 2025). The covariation of stable carbon and nitrogen isotopes is consistent with previous studies; as δ^15^N values become more positive δ^13^C values become more negative, with this being associated with increased influence of agriculture (Anderson and Cabana 2005). Areas with significant anthropogenic activities and low drainage are also known to have higher concentrations of methane in neighbouring waterbodies (Stanley et al. 2016; Borges et al. 2018; Essert et al. 2022). A relationship between sites with high methane concentrations and lower δ^13^C values in invertebrates has been well described in Europe (Essert et al. 2022; Olid et al. 2021; Pacioglu et al. 2022). Although not measured in this study, UK streams are commonly supersaturated in CH_4_ (Stanley et al. 2016; Sampson et al. 2019), thus it is highly plausible that variations in trout δ^13^C reflect stream microbial processes such as methane oxidation, carbon from which then flows through the stream food web to the upper trophic levels.

### 4.2 Pasture Effects on Stream δ^13^C Values

Our findings indicate that pastures are the land use with the most significant influence on δ^13^C values in trout fry in upland streams. This is in line with the current understanding that agricultural practices, particularly those involving livestock, are associated with several factors that affect methane production and oxidation (Chan and Parkin 2001; Borges et al. 2018; Jackson et al. 2020).

Livestock have been linked to increased sedimentation in streams through the trampling of soils and erosion of riverbanks, in small streams within Patagonia (Argentina) and Oregon (US) (Horak et al. 2019; Kauffman et al. 2022). Fine sediment deposition can be a governing factor in creating hotspots for microbial methane production and oxidation; sediment reduces pore size in the benthos, preventing flow from replenishing oxygen and other terminal electron acceptors, thus creating conditions favourable for methanogens (Bodmer et al. 2020). The link between these methane ‘hotspots’ and invertebrate MDC has been shown in lotic sediment banks, with MDC contributing up to 50% of the total carbon in some primary producers found within a sediment depositional bank in the Danube (Pacioglu et al. 2022). Although sediment deposits/banks are less likely to be as extensive in streams than in large rivers such as the Danube studied by Pacioglu et al. (2022), deposits in streams caused by the trapping of sediments in macrophyte beds have been shown to increase methane production (Sanders et al. 2007).

In addition to sedimentation rates, livestock can change the microbial community of neighbouring soils through the addition of OM, nutrients, and gut microbiota in their excrement (Praeg et al. 2014; Fritze et al. 2021). Organic matter adds additional substrate needed for methanogenesis (e.g., acetate and methyl compounds) (Conrad 2020) while gut microbiomes of ruminants such as cows (Praeg et al. 2014) and deer (Fritze et al. 2021) contain methanogenic taxa that can survive within the soil, thus increasing the abundance of methane producers. Additionally, the high nitrogen inputs, specifically ammonium associated with agriculture, increases methanotrophic activity within the soil, changing the community compositions and increasing oxidation rates (Chan and Parkin 2001; Praeg et al. 2014).

For most methane oxidation processes, aerobic conditions are required (Chan and Parkin 2001; Bodmer et al. 2020). Both waterlogging and OM inputs from livestock manure deplete the soil oxygen concentrations, reducing or inhibiting methane oxidation, which in turn can lead to higher CH_4_ concentrations and increases in emissions to the atmosphere and groundwater/runoff into streams (Cardoso et al. 2019). Coupling this with potential increased sedimentation and OM runoff from pastures can facilitate in‐stream methanogenesis, thus creating a ‘hotspot’ of CH_4_ concentrations (Bodmer et al. 2020). Streams are generally well oxygenated, and the combination of high O_2_ concentrations alongside high CH_4_ inputs provides ideal conditions for MOB (Nagler et al. 2020; Robison et al. 2022). Increases in MOB create a route for MDC to enter the food‐web from both the CO_2_ produced from CH_4_ oxidation being utilised for photosynthesis (Knoblauch et al. 2015; Grey 2016) and inclusion in the tissues of plants or from direct grazing of the methanogenic and methanotrophic microorganisms by invertebrates (Sampson et al. 2019; Pacioglu et al. 2022).

### 4.3 Low Drainage Soils Effects on Stream δ^13^C Values

Low drainage soils were shown to be the top correlate with low δ^13^C values in the trout. This furthers the link to methane derived carbon with low soil drainage known to increase the production of CH_4_ within the soil (Shukla et al. 2013; Praeg et al. 2014), which can be exported to streams through groundwater. Methane concentrations within streams have been linked to hydrological flows through neighbouring waterlogged soils such as in peatland, floodplains and adjacent wetlands (Stanley et al. 2016).

The areas categorised as high drainage in the Tweed are characterised by brown earths and humus‐iron podzols while the lower drainage areas tended towards types of gley soils and peat (noncalcareous gleys, peaty gleys, peaty gleyed podzols). Gley layers in soils have been recognised for increased methanogenesis (He et al. 2024). The displacement of air in the soil pores by water lowers available oxygen for decomposition, and the remaining terminal electron acceptors are readily used up. These conditions are favourable for methanogenesis but also reduce decomposition rates of organic matter (OM). This lack of decomposition can lead to increases in the amount of OM seeping into waterbodies (Abril and Borges 2019) and δ^13^C values for in‐stream dissolved organic carbon (DOC) have been found to match those of the organic matter (OM) and litter found within neighbouring waterlogged soils (Nkoue Ndondo et al. 2021). The increased accumulation of terrestrial OM within the stream benthos from neighbouring low drainage soils may in turn create in‐stream methane production within stream sediments (Romeijn et al. 2019; Berberich et al. 2020; Leng et al. 2021; Zhu et al. 2022).

The high concentrations of methane seen within streams (Stanley et al. 2016) can be attributed to both in‐stream production from influxes of organic matter and ground water inputs (Crawford et al. 2017). Higher stream dissolved methane concentrations lead to an increase in methane oxidation (Trimmer et al. 2015); in turn this would mean more MDC available to stream organisms and consumers of MOB, or more CO_2_ derived from CH_4_ oxidation (Grey 2016). Sampson et al. (2019) did not find a link between CH_4_ concentrations and δ^13^C values, but this may have been due to a limited range of variation in CH_4_ concentrations, with most streams being supersaturated. They did however find an effect of underlying geology, with chalk streams showing lower δ^13^C values in caddisfly. The sample sites within the Tweed catchment were mostly found on shale, a geology previously noted for having stream water with higher CH_4_ and lower CO_2_ concentrations, an indicator of anaerobic activity (Jones and Mulholland 1998). These findings from both systems highlight the potential importance of hydrology and groundwater flows in governing MDC availability. There likely are multiple pathways by which high MDC availability can occur, with the lower δ^13^C values found in the trout from catchments associated with low drainage soils highlighting a potential pathway through catchment soil processes.

### 4.4 Spatial Effects of Catchment

Models based on larger buffer sizes/extensive catchment areas explaining more of the variation in trout δ^13^C values suggest that the variance is not solely dependent on the catchment characteristics in the immediate area of sampling. This would suggest that groundwater draining from a wider area across the catchment may play a significant role in governing MDC abundance. The significance of low (and hence slow) soil drainage in combination with catchment characteristics in buffers covering areas further away from the stream on trout δ^13^C values could imply that increases in groundwater residency time and accumulation of organic matter may be a key factor in increasing MDC contributions to stream production. Longer groundwater residency has been linked to anaerobic conditions from microbial consumption of oxygen and other electron acceptors (Molofsky et al. 2013; McPhillips et al. 2014). Although the analysis shows a trend towards entire catchments explaining the most variability, it did not take into account specific flow paths of groundwater across the catchment area. Future analysis could examine more detailed flow paths in catchment areas to account for variation in the characteristics of the land which water is draining.

### 4.5 Invertebrate—Fish Isotopic Relationship

The strong correlation between trout and *Baetis* spp. δ^13^C values reflects the known strong trophic link between the two species (Kelly‐Quinn and Bracken 1990; Sánchez‐Hernández et al. 2011; Giller and Greenberg 2015). The trout's average δ^13^C enrichment of 2.53‰ compared to the *Baetis* is higher than the typical 1.3‰ ± 0.3‰ enrichment from fractionation when carbon is assimilated to consumer muscle tissues (McCutchan Jr et al. 2003). The increase in δ^13^C above the expected fractionation rate likely reflects that trout fry do not solely consume *Baetis*. *Baetis* are one of the most abundant taxa within these upland streams, and fit within the grazer/scraper functional feeding group (FFG), the same group as Goeridae and Glossosomatidae, taxa known to utilise MDC (Tuffin 2014; Sampson 2015; Sampson et al. 2019). Thus, *Baetis* δ^13^C values are likely to reflect those of other MDC‐utilising taxa in the streams, in addition to indicating that *Baetis* can utilise MDC, as has been suggested previously (Pearson 2015). Thus, the strong correlation between trout fry and *Baetis* strengthens the suggestion that the trout data presented here may reflect utilisation of MDC further down the food chain.

## 5 Conclusion

This study establishes a connection between low δ^13^C values in a stream top consumer and catchment characteristics with previously established links to elevated stream methane concentrations. The characteristics highlighted, low drainage soils and agricultural pastures, are consistent with those identified by previous work as enhancing methane inputs to streams, but the link between these and the extent of methane incorporation into food webs as reflected in stable carbon isotope values has not previously been demonstrated. Whilst we also demonstrate a link here between the low carbon isotope values of the top consumer and lower trophic levels, the details of the mechanisms and pathways by which incorporation and transfer of MDC through food webs occur remain unclear and warrant further investigation at the level of microbial communities upwards and considering the role of other environmental parameters.

Our findings have implications for catchment and stream management strategies in relation to stream carbon budgets and flows. There is a well‐established link between land use and levels of methane entering streams, but the influence on methane oxidation and thus retention in stream biomass is less well studied. Stream carbon budgets should take into consideration the in‐stream processes of oxidation and the slowing/reduction of emissions of methane through sequestration into the tissues of organisms.

## Author Contributions

**Michael Hinchliffe:** conceptualization (equal), data curation (equal), formal analysis (equal), investigation (equal), methodology (equal), visualization (equal), writing – original draft (equal), writing – review and editing (equal). **Aimeric Blaud:** conceptualization (equal), funding acquisition (equal), investigation (equal), methodology (equal), supervision (equal), writing – review and editing (equal). **Peter Gilbert:** conceptualization (equal), funding acquisition (equal), investigation (equal), methodology (equal), supervision (equal), writing – review and editing (equal). **Rona McGill:** formal analysis (equal), methodology (equal), writing – review and editing (equal). **Kenny Galt:** investigation (equal), methodology (equal), writing – review and editing (equal). **Robert A. Briers:** conceptualization (equal), data curation (equal), formal analysis (equal), funding acquisition (equal), investigation (equal), methodology (equal), project administration (equal), supervision (equal), visualization (equal), writing – original draft (equal), writing – review and editing (equal).

## Funding

This work was supported by Natural Environment Research Council (NE/S007342/1) and the National Environmental Isotope Facility (NEIF/2480).

## Conflicts of Interest

The authors declare no conflicts of interest.

## Supporting information


**Table S1:** Mean length, weight δ^13^C and δ^15^N from the 10 trout fry sampled at each location. Standard error has been included for the average length and weight. Also given is the mean δ^13^C for *Baetis* where sampled.
**Table S2:** Summary table of all candidate linear models compared used in model selection. CF—Coniferous forest, MH—Moors & Heathland, Past—Pastures, TWS—Transitional woodland shrub, LD—Low drainage, MD—Mixed drainage, HD—High Drainage, Alt—Average altitude, Slope—Average slope, The confidence set is formed of the two models highlighted in bold, selection was based on ΔAIC < 2.
**Figure S3:** *Baetis* δ^13^C values for each of the 26 sites sampled. δ^13^C values ranged from −43.3‰ to −26.3‰ showing significant variation between sites (ANOVA: *F*_(25,20)_ = 14.81, *p* < 0.0001).

## Data Availability

Data and code associated with this study are available in a Github repository (https://github.com/robbriers/trout_carbon_isotopes).
