# Supplementary material for: Catchment Influences on Carbon Stable Isotope Variation in Trout; Might It Be Methane?
Source: Ecol Evol. 2026 Apr 29;16(5):e73554. doi: 10.1002/ece3.73554 (PMC13126239; doi:10.1002/ece3.73554)
Supplement: Supplementary file 1 — Table S1: Mean length, weight δ13C and δ15N from the 10 trout fry sampled at each location. Standard error has been included for the average length and weight. Also given is the mean δ13C for Baetis where sampled. Table S2: Summary table of all candidate linear models compared used in model selection. CF—Coniferous forest, MH—Moors & Heathland, Past—Pastures, TWS—Transitional woodland shrub, LD—Low drainage, MD—Mixed drainage, HD—High Drainage, Alt—Average altitude, Slope—Average slope, The confidence set is formed of the two models highlighted in bold, selection was based on ΔAIC < 2. Figure S3: Baetis δ13C values for each of the 26 sites sampled. δ13C values ranged from −43.3‰ to −26.3‰ showing significant variation between sites (ANOVA: F (25,20) = 14.81, p < 0.0001). [file ECE3-16-e73554-s001.docx]

**Catchment influences on carbon isotope variation in trout; might it be methane?**

Michael Hinchliffe^a,b^, Aimeric Blaud^a,b^, Peter Gilbert^c,d^, Rona McGill^e^, Kenny Galt^f†^, Robert A Briers^a,b^

Corresponding author: Robert A Briers [r.briers@napier.ac.uk](mailto:r.briers@napier.ac.uk)

^a^ Edinburgh Napier University, School of Applied Sciences, Edinburgh Napier University, Edinburgh, EH11 4BN, UK.

^b^ Centre for Conservation and Restoration Science, Edinburgh Napier University, Edinburgh, EH11 4BN, UK.

^c^ University of Highlands and Islands, Environmental Research Institute, Thurso, KW14 7JD, UK.

^d^ Royal Society for the Protection of Birds (RSPB), Etive House, Inverness, IV2 3BW

^e^ Stable Isotope Ecology Lab, Natural Environment Isotope Facility, Scottish Universities Environmental Research Centre, East Kilbride, G75 0QF, UK.

^f^ Tweed Foundation, Drygrange Steading, Melrose, Roxburghshire, TD6 9DJ

† Current address: Galloway Fisheries Trust, Newton Stewart, Wigtownshire, DG8 6ND

Michael Hinchliffe – [m.hinchliffe@napier.ac.uk](mailto:m.hinchliffe@napier.ac.uk)

Aimeric Blaud – [a.blaud@napier.ac.uk](mailto:a.blaud@napier.ac.uk)

Peter Gilbert – [peter.gilbert@uhi.ac.uk](mailto:peter.gilbert@uhi.ac.uk)

Rona A. R. McGill – [rona.mcgill@glasgow.ac.uk](mailto:rona.mcgill@glasgow.ac.uk)

Kenny Galt – [kenny@gallowayfisheriestrust.org](mailto:kenny@gallowayfisheriestrust.org)

Robert Briers – [r.briers@napier.ac.uk](mailto:r.briers@napier.ac.uk)

**Table S1.** Mean length, weight δ^13^C and δ^15^N from the 10 trout fry sampled at each location. Standard error has been included for the average length and weight. Also given is the mean δ^13^C for *Baetis* where sampled.

| ***Site*** | ***Mean Length (SE)*** | ***Mean Weight (SE)*** | ***Mean Trout δ^13^C*** | ***Mean Baetis δ^13^C*** | ***Mean Trout δ^15^N*** | ***Mean Baetis δ^15^N*** |
| --- | --- | --- | --- | --- | --- | --- |
| *G01* | 4.44 ± 0.08 | 1.05 ± 0.12 | -31.48 | -36.81 | 11.54 | 8.03 |
| *G02* | 4.71 ± 0.21 | 1.29 ± 0.19 | -29.96 |  | 7.45 |  |
| *G03* | 3.89 ± 0.13 | 0.72 ± 0.07 | -28.81 |  | 8.34 |  |
| *G04* | 4.17 ± 0.18 | 1.02 ± 0.14 | -26.53 | -28.17 | 9.25 | 3.47 |
| *G05* | 4.65 ± 0.27 | 1.23 ± 0.13 | -29.67 |  | 8.64 |  |
| *G06* | 4.43 ± 0.17 | 1.17 ± 0.12 | -28.59 | -29.32 | 7.65 | 2.72 |
| *G07* | 4.16 ± 0.13 | 0.93 ± 0.08 | -29.08 |  | 8.08 |  |
| *G08* | 4.06 ± 0.11 | 0.87 ± 0.08 | -26.70 |  | 7.91 |  |
| *G09* | 4.42 ± 0.14 | 1.21 ± 0.14 | -31.82 |  | 10.97 |  |
| *G10* | 5.35 ± 0.21 | 1.90 ± 0.21 | -31.92 |  | 9.63 |  |
| *G11* | 4.47 ± 0.15 | 1.14 ± 0.13 | -30.81 | -34.10 | 10.93 | 7.61 |
| *G12* | 4.44 ± 0.15 | 1.13 ± 0.10 | -30.18 |  | 10.77 |  |
| *G13* | 5.15 ± 0.22 | 1.96 ± 0.23 | -31.99 | -37.87 | 10.30 | 6.97 |
| *G14* | 4.61 ± 0.16 | 1.18 ± 0.12 | -29.92 |  | 8.06 |  |
| *G15* | 4.83 ± 0.07 | 1.34 ± 0.08 | -29.30 |  | 8.68 |  |
| *G16* | 5.32 ± 0.15 | 1.67 ± 0.16 | -32.12 |  | 7.88 |  |
| *G17* | 5.00 ± 0.13 | 1.52 ± 0.11 | -30.38 |  | 8.55 |  |
| *G18* | 5.16 ± 0.17 | 1.88 ± 0.17 | -28.99 |  | 8.49 |  |
| *G20* | 5.57 ± 0.21 | 2.13 ± 0.25 | -26.46 |  | 8.31 |  |
| *G21* | 5.76 ± 0.16 | 2.36 ± 0.24 | -26.83 | -26.80 | 9.02 | 3.64 |
| *G22* | 5.01 ± 0.22 | 1.67 ± 0.21 | -24.88 |  | 10.33 |  |
| *U01* | 5.69 ± 0.14 | 2.23 ± 0.16 | -30.72 | -35.67 | 9.84 | 6.65 |
| *U02* | 4.63 ± 0.24 | 1.31 ± 0.21 | -30.79 | -31.03 | 9.10 | 6.31 |
| *U03* | 5.58 ± 0.14 | 2.11 ± 0.15 | -29.29 |  | 6.52 |  |
| *U05* | 5.68 ± 0.13 | 2.28 ± 0.15 | -29.91 | -33.67 | 7.88 | 3.71 |
| *U07* | 6.22 ± 0.12 | 3.64 ± 0.21 | -32.01 | -33.74 | 7.13 | 2.85 |
| *U09* | 5.06 ± 0.17 | 1.65 ± 0.18 | -28.26 |  | 7.13 |  |
| *U11* | 6.72 ± 0.18 | 3.81 ± 0.32 | -28.55 |  | 6.51 |  |
| *U12* | 5.87 ± 0.12 | 2.36 ± 0.12 | -26.25 | -27.75 | 6.82 | 2.24 |
| *U13* | 5.73 ± 0.16 | 2.33 ± 0.18 | -28.05 | -29.26 | 7.80 | 3.69 |
| *U14* | 5.80 ± 0.10 | 2.63 ± 0.13 | -27.61 |  | 6.97 |  |
| *U16* | 5.49 ± 0.24 | 2.42 ± 0.27 | -26.46 | -27.73 | 7.97 | 3.24 |
| *U17* | 5.66 ± 0.25 | 2.09 ± 0.24 | -30.00 | -33.29 | 8.06 | 8.32 |
| *U18* | 5.52 ± 0.18 | 2.01 ± 0.18 | -30.26 |  | 6.91 |  |
| *U19* | 5.78 ± 0.16 | 2.46 ± 0.19 | -28.58 | -32.10 | 10.48 | 6.73 |
| *U21* | 5.22 ± 0.14 | 1.78 ± 0.15 | -30.48 | -36.91 | 9.78 | 8.93 |
| *U22* | 5.40 ± 0.23 | 2.03 ± 0.24 | -30.68 |  | 11.49 |  |
| *U23* | NA | NA | -36.86 | -41.72 | 12.49 | 9.78 |
| *U24* | 5.11 ± 0.11 | 1.91 ± 0.15 | -31.63 | -33.32 | 10.04 | 5.39 |
| *U25* | 5.14 ± 0.15 | 1.80 ± 0.18 | -27.72 | -30.39 | 7.89 | 4.12 |
| *U26* | 6.42 ± 0.21 | 3.49 ± 0.33 | -33.60 |  | 11.61 |  |
| *U27* | 5.02 ± 0.21 | 1.71 ± 0.20 | -27.81 |  | 10.22 |  |
| *U28* | 6.10 ± 0.15 | 2.79 ± 0.22 | -30.00 |  | 10.44 |  |
| *U30* | 4.25 ± 0.12 | 1.00 ± 0.09 | -30.62 | -33.87 | 7.06 | 1.44 |
| *U31* | 4.71 ± 0.18 | 1.28 ± 0.10 | -30.16 |  | 7.91 |  |
| *U32* | 4.40 ± 0.16 | 0.97 ± 0.11 | -27.31 |  | 5.98 |  |
| *U33* | 3.78 ± 0.09 | 0.63 ± 0.05 | -28.15 |  | 7.67 |  |
| *U34* | 4.37 ± 0.15 | 0.98 ± 0.11 | -29.11 | -31.72 | 7.25 | 2.07 |
| *U35* | 4.97 ± 0.18 | 1.57 ± 0.16 | -28.67 |  | 7.77 |  |
| *U37* | 5.19 ± 0.15 | 1.73 ± 0.15 | -30.06 |  | 7.96 |  |

**Table S2.** Summary table of all candidate linear models compared used in model selection. CF – Coniferous forest, MH – Moors & Heathland, Past – Pastures, TWS – Transitional woodland shrub, LD – Low drainage, MD – Mixed drainage, HD – High Drainage, Alt – Average altitude, Slope – Average slope, The confidence set is formed of the two models highlighted in bold, selection was based on ΔAIC < 2.

| Model Rank | Models | Intercept | CF | MH | Pa | Peat | TWS | LD | MD | HD | Alt | Slope | LD*Pa | df | logLik | AICc | delta | weight |
| --- | --- | --- | --- | --- | --- | --- | --- | --- | --- | --- | --- | --- | --- | --- | --- | --- | --- | --- |
| **1** | **Low drainage** | -28.5 |  |  |  |  |  | -.093 |  |  |  |  |  | 3 | -96.1 | 198.6 | 0.0 | 0.32 |
| **2** | **Low drainage + Pastures** | -28.4 |  |  | -.014 |  |  | -.080 |  |  |  |  |  | 4 | -95.4 | 199.6 | 1.0 | 0.19 |
| 3 | Low drainage + Pastures + Altitude | -25.9 |  |  | -.024 |  |  | -.092 |  |  | -.006 |  |  | 5 | -94.7 | 200.7 | 2.1 | 0.11 |
| 4 | Low drainage + Altitude | -28.1 |  |  |  |  |  | -.096 |  |  | -.001 |  |  | 4 | -96.0 | 201.0 | 2.3 | 0.10 |
| 5 | Low drainage + Pastures + Slope | -27.7 |  |  | -.018 |  |  | -.087 |  |  |  | -.061 |  | 5 | -94.8 | 201.0 | 2.4 | 0.10 |
| 6 | Low drainage + Pastures + Altitude + Slope | -25.8 |  |  | -.025 |  |  | -.096 |  |  | -.005 | -.049 |  | 6 | -94.4 | 202.6 | 4.0 | 0.04 |
| 7 | Pastures | -28.8 |  |  | -.039 |  |  |  |  |  |  |  |  | 3 | -102.0 | 210.6 | 11.9 | 0.00 |
| 8 | Pastures + High drainage | -29.4 |  |  | -.046 |  |  |  |  | .015 |  |  |  | 4 | -101.2 | 211.4 | 12.7 | 0.00 |
| 9 | Pastures + Altitude | -30.2 |  |  | -.031 |  |  |  |  |  | .003 |  |  | 4 | -101.8 | 212.5 | 13.8 | 0.00 |
| 10 | Pastures + Mixed drainage | -28.7 |  |  | -.040 |  |  |  | -.004 |  |  |  |  | 4 | -102.0 | 212.9 | 14.2 | 0.00 |
| 11 | Pastures + High drainage + Altitude | -31.5 |  |  | -.036 |  |  |  |  | .017 | .005 |  |  | 5 | -100.8 | 212.9 | 14.3 | 0.00 |
| 12 | Pastures + Mixed drainage + Altitude | -31.5 |  |  | -.036 |  |  |  |  | .017 | .005 |  |  | 5 | -100.8 | 212.9 | 14.3 | 0.00 |
| 13 | Altitude | -33.4 |  |  |  |  |  |  |  |  | .010 |  |  | 3 | -103.5 | 213.5 | 14.8 | 0.00 |
| 14 | Pastures + Mixed drainage + High drainage | -29.7 |  |  | -.045 |  |  |  | .010 | .017 |  |  |  | 5 | -101.2 | 213.6 | 15.0 | 0.00 |
| **15** | **Global** | **-26.4** | **.003** |  | **-.023** | **.000** | **.096** | **-.101** | **.003** | **.003** | **-.004** | **-.069** |  | **11** | **-93.2** | **215.3** | **16.6** | **0.00** |
| 16 | Transitional woodland scrub + Altitude | -33.5 |  |  |  |  | .065 |  |  |  | .010 |  |  | 4 | -103.2 | 215.3 | 16.6 | 0.00 |
| 17 | Pastures + Mixed drainage + High drainage + Altitude | -31.9 |  |  | -.034 |  |  |  | .010 | .020 | .005 |  |  | 6 | -100.7 | 215.3 | 16.7 | 0.00 |
| 18 | Altitude + Peat | -33.7 |  |  |  | -.005 |  |  |  |  | .011 |  |  | 4 | -103.4 | 215.6 | 17.0 | 0.00 |
| 19 | Moors & Heathland | -30.1 |  | .019 |  |  |  |  |  |  |  |  |  | 3 | -105.4 | 217.4 | 18.7 | 0.00 |
| 20 | Transitional woodland scrub + Coniferous forest + Altitude | -33.5 | .004 |  |  |  | .050 |  |  |  | .010 |  |  | 5 | -103.2 | 217.6 | 19.0 | 0.00 |
| 21 | Slope | -30.4 |  |  |  |  |  |  |  |  |  | .105 |  | 3 | -105.9 | 218.3 | 19.7 | 0.00 |
| 22 | Low drainage + Slope | -30.4 |  |  |  |  |  |  |  |  |  | .105 |  | 3 | -105.9 | 218.3 | 19.7 | 0.00 |
| 23 | Mixed drainage | -29.9 |  |  |  |  |  |  | .028 |  |  |  |  | 3 | -106.3 | 219.2 | 20.6 | 0.00 |
| 24 | Peat | -29.6 |  |  |  | .011 |  |  |  |  |  |  |  | 3 | -106.8 | 220.1 | 21.5 | 0.00 |
| 25 | High drainage + Slope | -30.2 |  |  |  |  |  |  |  | -.003 |  | .101 |  | 4 | -105.9 | 220.6 | 21.9 | 0.00 |
| 26 | Coniferous forest | -29.6 | .010 |  |  |  |  |  |  |  |  |  |  | 3 | -107.1 | 220.7 | 22.1 | 0.00 |
| 27 | High drainage | -29.1 |  |  |  |  |  |  |  | -.007 |  |  |  | 3 | -107.2 | 221.0 | 22.4 | 0.00 |
| 28 | Transitional woodland scrub | -29.5 |  |  |  |  | .040 |  |  |  |  |  |  | 3 | -107.3 | 221.2 | 22.5 | 0.00 |
| 29 | High drainage + Peat | -29.7 |  |  |  | .011 |  |  |  | .001 |  |  |  | 4 | -106.8 | 222.5 | 23.8 | 0.00 |
| 30 | High drainage + Peat + Slope | -30.6 |  |  |  | .009 |  |  |  | .003 |  | .095 |  | 5 | -105.6 | 222.5 | 23.9 | 0.00 |
| 31 | Transitional woodland scrub + Coniferous forest | -29.6 | .010 |  |  |  | .006 |  |  |  |  |  |  | 4 | -107.1 | 223.1 | 24.5 | 0.00 |


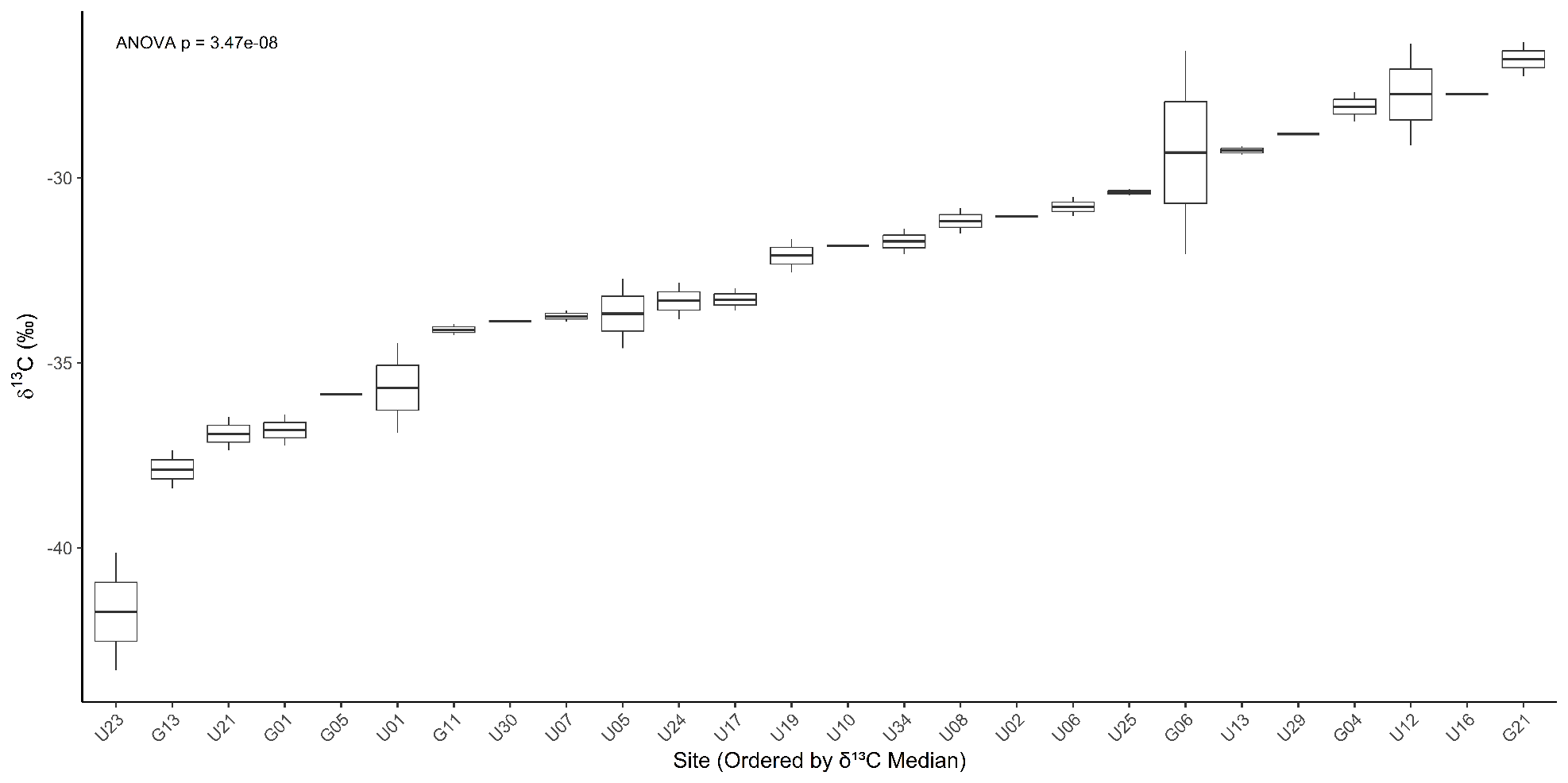


**Figure S3.** *Baetis* δ^13^C values for each of the 26 sites sampled. δ^13^C values ranged from -43.3‰ to -26.3‰ showing significant variation between sites (ANOVA: *F*_(25,20)_ = 14.81, *p* < 0.0001).
